# Building an intentional and impactful summer research experience to increase diversity in mental health research

**DOI:** 10.1038/s41386-022-01461-8

**Published:** 2022-10-12

**Authors:** Oluwarotimi O. Folorunso, Karen Burns White, Yanaira Alonso-Caraballo, Genevieve P. Nowicki, Elizabeth A. Olson, Diego A. Pizzagalli, William A. Carlezon, Kerry J. Ressler, Elena H. Chartoff

**Affiliations:** 1 Department of Psychiatry, Harvard Medical School, Boston, MA USA; 2 Basic Neuroscience Division, McLean Hospital, Belmont, MA USA; 3 Dana-Farber/Harvard Cancer Center, Boston, MA USA; 4 Center for Depression, Anxiety and Stress Research, McLean Hospital, Belmont, MA USA

**Keywords:** Diseases of the nervous system, Psychiatric disorders

Diversity in health care and research is associated with improvements in health care access and outcomes [1]. Historical and structural inequalities have led to disparities in mental health outcomes in the U.S., and representation in the mental health workforce is critically needed to address health disparities [2]. Compared to the general population, people from minoritized racial and ethnic groups seek and receive fewer mental health services and are significantly underrepresented in mental health research studies. Rates of mental health conditions are similar among those who are minoritized due to race or ethnicity and those who are not, but reduced rates of treatment and research access and engagement result in worse mental health outcomes for minoritized people [3]. A major systemic factor contributing to these disparities is a lack of diverse neuroscientists, clinical psychologists, and psychiatrists, resulting in a paucity of multiculturally competent research and clinical care [1]. For example, increased representation in research could improve our understanding of differences in treatment effects to provide better mental health care.

A lack of racial and ethnic diversity across these fields has been repeatedly documented. According to the National Science Foundation, the following racial and ethnic groups have been shown to be underrepresented in the fields of biomedical, clinical, behavioral, and health services research: Blacks or African Americans, Hispanics or Latinos, American Indians or Alaska Natives, Native Hawaiians and other Pacific Islanders [4]. This dearth of diversity contributes to biases in mental health research [5], reduces trust, accessibility, and participation in clinical studies, and acts as a roadblock to appropriate care between caregivers and their patients. For example, according to the Society for Neuroscience (SfN) Departments & Program Survey (2016–2017) [6], 6% of predoctoral students, 3% of postdoctoral fellows, and 1% of faculty in neuroscience departments in the U.S. were Black in 2016. These percentages are similar for other persons excluded because of their ethnicity or race (*PEER*) [7, 8] groups (Fig. 1). In 2019, 12% of psychologists belonged to these underrepresented groups (4% Black, 7% Hispanic, 2% ‘Other’ (i.e., American Indian, Alaska Native, Native Hawaiian/other Pacific Islanders, and other races and those reporting two or more races) [9]. Within psychiatry, 16% of residents, 9% of faculty, and 10% of practicing physicians were from PEERs (including Black, Hispanic, American Indian/Alaska Native, Native Hawaiian/other Pacific Islanders) [10]. Despite Black people constituting 14% of the US population, only 4.4% of psychiatrists are Black. Furthermore, representation among PEER scientists falls precipitously over an academic career trajectory (Fig. 1).

**Fig. 1:**
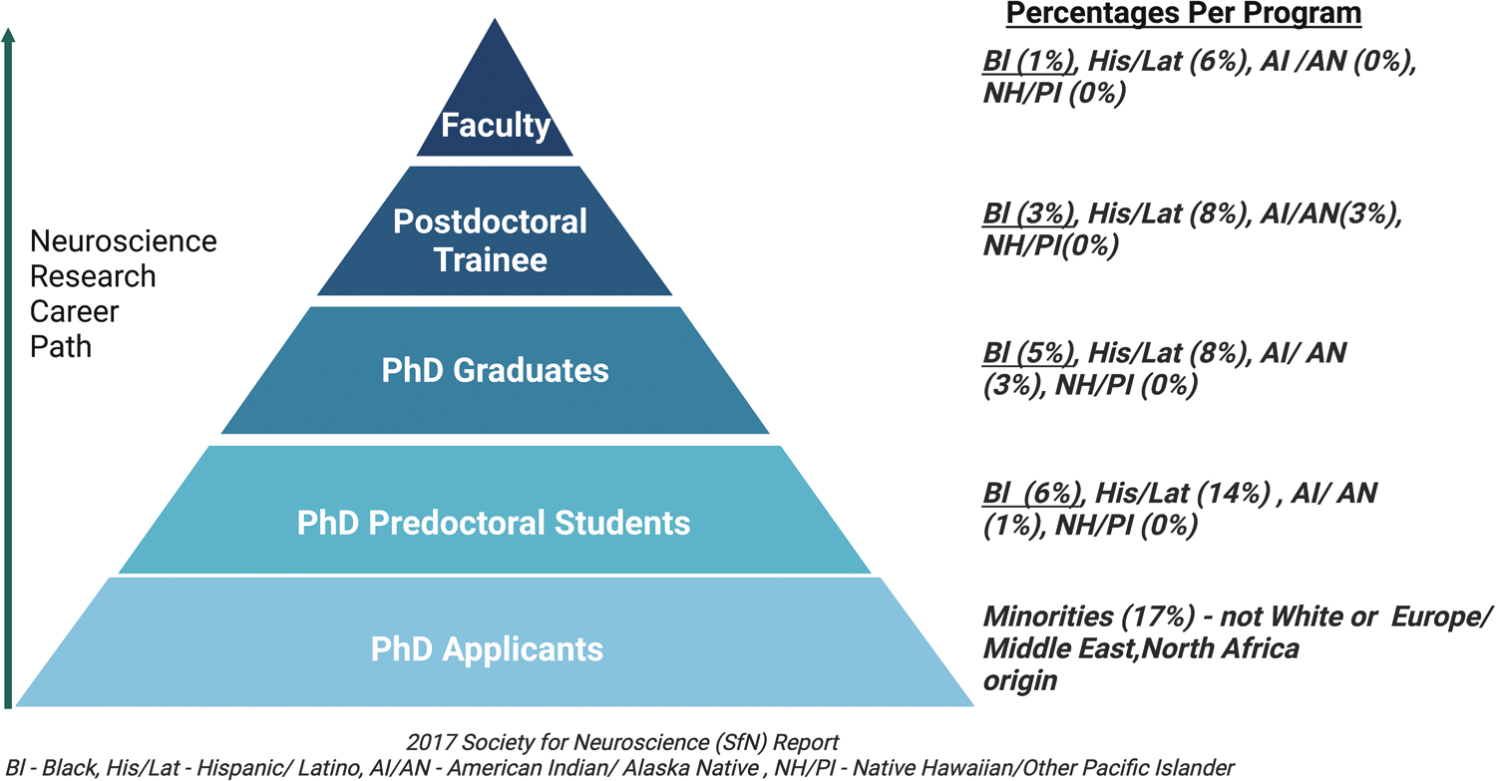
2017 Society of Neuroscience (SfN) Report showing the average percentage of racial/ethnic minorities per Neuroscience Programs in the US. (For PhD Applicants, a more detailed breakdown of race and ethnicity was not provided). Figure was created with Biorender.

These grim statistics highlight a vital need for recruitment and retention of PEERs in neuroscience, psychology, and psychiatry. This can be addressed, in part, in at least three ways: (1) provide paid research opportunities for college students at research institutions, (2) provide consistent and personalized mentorship, and (3) build PEER communities within the sciences to support and advocate for equity and inclusion, as well as to address the leakage of PEERs along their career trajectory [11]. We emphasize *paid* research opportunities because financial barriers have historically prevented and discouraged PEER students from participating in summer research programs [12].

In the U.S., intersectional disparities in education attainment and quality exist by race, ethnicity, and socioeconomic status (SES) [13]. While ameliorating these inequities requires purposeful and sustained cooperation across disciplines, agencies, and communities, one component relevant to the mental health field includes creating long-lasting partnerships between academic research institutions, K-12 classrooms, and higher-education institutions, particularly community colleges, that offer opportunities for PEER students to gain familiarity and participate in mental health research in developmentally appropriate capacities throughout their education [9].

This perspective on creating intentional summer programs highlights some of the lessons learned from the McLean Mental Health Research Summer Program (MMHRSP), which was developed in 2021 at McLean Hospital. McLean is the primary psychiatric teaching hospital of Harvard Medical School, and it maintains the largest neuroscience research program of any affiliated hospital, department, or school of Harvard University. Approximately 220 investigators work on over 400 funded research projects at McLean. MMHRSP’s overarching goal is to increase PEER representation in mental health research by providing hands-on, *paid* research experiences, *committed* long-term mentorship, academic networking, and K-12 science outreach opportunities.

## Keys to an effective summer research experience for PEER undergraduate trainees

### Mentorship

One of the most significant factors in fostering students’ connections to neuroscience research is a long-lasting mentor-trainee relationship [14]. The career trajectories of PEER trainees are positively impacted by mentors with racial and cultural awareness, a positive attitude, and the ability to effectively interact with individuals from diverse backgrounds in science education and research [15, 16]. To that end, our program implemented evidence-based seminars on cross-cultural mentoring to Principal Investigators (PIs) and mentors before the summer research experience [16]. We found that our pre-summer seminars for mentors helped to ensure that initial meetings between mentors and trainees set the tone for success. Further, we developed a learning agreement between the mentor and trainee that delineated what is to be learned, how it will be learned, and how success can be evaluated. We encouraged the PIs to play an active role in the mentoring—even if they were not directly supervising the trainee—through regularly scheduled one-on-one meetings. Importantly, before the MMHRSP began, we met with the mentors to emphasize that the educational and personal backgrounds of trainees would vary. While the MMHRSP expectations were rigorous and equitable, we highlighted that the process by which learning occurs would be different for each student, and that trainee self-confidence and mutual trust would be built through constructive verbiage and tone, as well as conversations about mentee and mentor backgrounds, career interests and goals, and challenges encountered [15]. A well-received component of the MMHRSP was the pairing of trainees with secondary PEER mentors at other institutions who met with their mentee once per month, provided career advice, and connected trainees to their own professional network.

“*..(it) was a healthy and very supportive lab, which I did not expect. I grew in my understanding of science and what it is to be a scientist. The work was exciting, and [my coworkers] were very inclusive in lab meeting discussions.*” – MMHRSP Trainee.

### Scientific community

Good scientific networks strengthen the scientific identity of PEER trainees [17]. We intentionally organized professional development workshops, panels, and seminars led by PEER scientists. By sharing their educational and career journeys, these scientists helped trainees see a similar future for themselves through the lens of the PEER leaders. To promote community during the summer program, we held weekly “check-ins” and hosted social gatherings where trainees discussed research progress and developed long-lasting connections with one another. We also leveraged popular social media apps (e.g. Discord, Instagram, Twitter) to keep in touch with the trainees both during and after the program. This allowed us to easily provide continued support and follow the post-graduation path of each trainee. We highlighted the achievements of the trainees on our social media, attended graduations, and regularly communicated with past trainees through Discord groups. Past trainees have been invited to serve on the Program Committee and have been peer mentors for new trainees. To inspire the next generation of diverse mental health researchers, MMHRSP trainees engaged in K-12 scientific outreach, including organizing virtual high school visits and a community brain fair that highlighted their summer research experience at McLean.

### Measuring success

Highly structured positive research experiences impact the interest of PEER undergraduates in science, technology, engineering, math, and medicine (STEMM) careers [18]. Our first-year trainees were first-generation, non-traditional, from rural communities and/or single-parent household students. Over 70% of trainees were from low-income backgrounds. There was a 3:1 ratio of females to males. 43% were mixed race (black/white/Hispanic or Latino), 43% Black or African American, and 14% were Hispanic or Latino/a/e. The MMHRSP assess both short-term and long-term outcomes. Our short-term outcomes include measuring the:

1. Percentage of students who maintain research contact with their mentor after the conclusion of the program. Since the MMHRSP catered to Massachusetts residents, we strongly encouraged mentees to continue working in the lab during the school year for academic credit or thesis work or as paid research assistants. About half of our past trainees worked in their summer labs during the school year.
2. Percentage of students submitting conference abstracts. Approximately halfway through the summer experience, we encouraged mentors to work with their trainees to submit scientific abstracts and travel awards to a conference (e.g., Society for Advancement of Chicanos/Hispanics and Native Americans in Science [SACNAS] or the Annual Biomedical Research Conference for Minoritized Scientists [ABRCMS]). These conferences occur in the fall, and the application process strengthens the mentor-trainee connection. Last year, our trainees received travel and poster awards. They reported that the experience of attending these conferences and being surrounded by other outstanding PEER scientists helped them to challenge imposter syndrome and expand their scientific networks [17].
3. Extent to which trainees report excitement about exploring careers in mental health research on the program surveys. MMHRSP trainees reported enthusiasm and confidence in exploring mental health careers.
4. Percentage of students who apply for research assistant or postbaccalaureate positions and explore graduate or MD-PhD programs. We connected our trainees with research opportunities, internships, and research assistant (RA) positions. About half of the MMHRSP trainees are working as RAs post-program.

Long-term outcomes include assessing the percentages of trainees who pursue: 1) graduate school in neuroscience or branches of psychology or medical school; 2) for medical students, specialization in a field of neuroscience (neurology, psychiatry). 3) obtaining postdoctoral fellowships, faculty, and/or clinical positions in neuroscience-related fields [19]. As part of the professional development series, trainees interacted with graduate and MD-PhD program directors, postdocs, graduate and medical students, and scientific society officers. To broaden the program’s reach, panels were open to all applicants to the program and trainees from other summer programs. Our past trainees are applying to graduate programs in the 2022-2023 and 2023-2024 cycle, and non-program trainees are also applying to graduate school through resources provided by the program. At the end of MMHRSP, trainees had a draft of their CVs and personal statements, oral and poster presentations, moderated academic panels and participated in outreach.

*“I was able to gain insight on the experience of working in the research field, how to apply to graduate schools, and finding ways in which I can advance my career in research by applying for outside events such as seminars and conferences. I was also placed with an amazing PI who worked one on one with me with reviewing my CV, gaining more knowledge on the research I was specifically working on in the summer and even helping me with applications to become a Research Assistant to gain more experience in research.*” – MMHRSP Trainee.

## Creating a mental health summer research program

### Program organizing committee

**Fig. 2:**
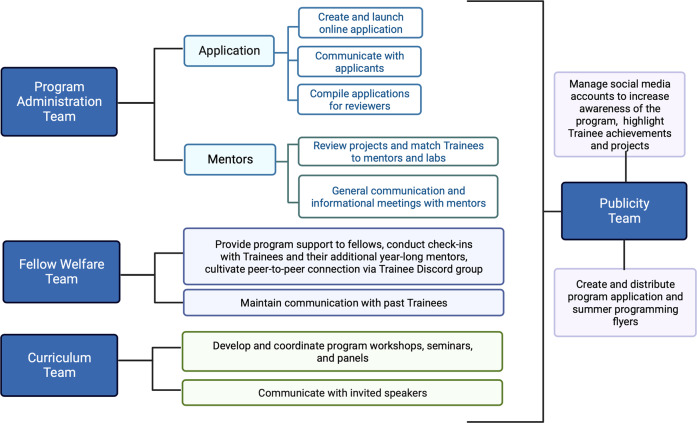
Organization of the program committee including the program administration, fellow welfare, curriculum, and publicity teams, and their respective general responsibilities. Figure was created with Biorender.

We encourage programs to assemble a diverse, on-site Program Committee that organizes and runs the program (Fig. 2) and an off-site Advisory Board to provide biannual feedback. The MMHRSP committee consists of PIs, postdoctoral fellows, postbaccalaureate research assistants, administrative staff, and one past MMHRSP trainee to provide peer perspectives. The Advisory Board consists of well-established PIs in the mental health field and other individuals invested in creating pathways to success for PEER undergraduates. To maximize the contributions of the Advisory Board, the Program Committee meets with members at the beginning and end of each summer program. The Advisory Board is also instrumental in promoting and advocating for the MMHRSP and its graduates.

### Funding

The NIH is increasingly committed to actions that enhance diversity at all levels of training. As stated in their Recruitment and Retention policy, “A recruitment plan should be developed by principal investigators, faculty members and senior leaders to identify strategies to enhance the pool of applicants for all programs”. There are multiple funding mechanisms designed to promote diversity in neuroscience research. However, is it initially hard to be competitive for these NIH initiatives. Given this, we turned to McLean’s Development Office and Division leaders for philanthropic and institutional support to launch the MMHRSP. Working closely with the Development Office, we secured over $60,000 for our first year. Critically, we budgeted to provide living-wage stipends to the PEER trainees. For our 10-week program in 2021, we paid each trainee $6,000, in addition to compensation for transit and lunch. Conscious of the “URM tax”, we also provided honoraria to our speakers and workshop facilitators for their diversity, equity, and inclusion efforts [20].

### Program curriculum

In addition to the primary research experience, the MMHRSP includes scientific education, professional development, networking activities, social gatherings, and outreach activities. Good resources for creating a curriculum include the Entering Research curriculum [21], the Science of Effective Mentoring report, and the Center for the Improvement of Mentored Experiences in Research (CIMER) workshops (https://cimerproject.org). Professional development workshops should address critical thinking and presentation skills as well as strategies to manage imposter syndrome and enhance cross-cultural communication. Our curriculum includes panels of neuroscience and clinical graduate program directors, racially and culturally diverse graduate students, and successful, mid-career PEER individuals in mental health careers. We leveraged the convenience afforded by videoconferencing to host people from all over the country to expand trainees’ networks. Finally, PEER workshop leaders, graduate students, and keynote speakers were compensated with an honorarium and sent Workshop and Mentoring Evaluation results for their teaching/DEI portfolio.

### MMHRSP advertising

The Program Committee is thoughtful about how we advertise and select our trainees. Every diversity-enhancing program is different, and our overarching goal is to provide a robust research and professional development experience to PEER students who might otherwise not have such a transformative experience. We intentionally prioritize outreach to community colleges, local minority student groups, and 4-year universities with neuroscience and psychology departments that do not have extensive research opportunities. We encourage programs to advertise at Minority Serving Institutions, Historically Black Colleges, and Universities (HBCUs), and Native American, Black, and Hispanic Student Groups.

### Choosing MMHRSP trainees

The Program Committee emphasizes to reviewers that selection criteria should be based primarily on the applicants’ interest and motivation, enthusiasm for research/science, the trainee’s resilience in overcoming adversity, and finally, the degree to which our program could help a trainee’s career. Our application elicits this information in several ways including through the Research Interests and Personal Statement sections, and one to two recommendation letters from science professors or advisors. The MMHRSP emphasizes in our application that the letters should address the importance of our Program to students and their enthusiasm and academic potential. We are rigorous to ensure that factors such as Grade Point Average (GPA) and prior research experience do not unintentionally exclude students who may benefit the most from our Program. To that end, while our application requests academic transcripts and GPA, we have an open-ended response section for students to explain whether they feel their GPA reflects their potential. The Program Committee chooses a diverse array of reviewers (e.g., research assistants, graduate students, postdoctoral fellows, and principal investigators) to avoid unintended biases. Each application is reviewed by three individuals, and each member of the Program Committee is assigned 8-10 applications and asked to choose their top two choices. The Program Committee then convenes for several hours to discuss the top-ranked choices and select the trainees. The MMHRSP emphasizes intersectionality throughout the application process and program, inviting applications to self-report additional marginalized identities such as gender identity, sexual orientation, SES, and disability status, and takes these into consideration when selecting trainees.

### Matching trainees to laboratories

The summer research experience should promote active involvement of both mentors and trainees through required deliverables. Historically, poor experiences with mentorship and environment, rather than the research per se, deter PEER students from pursuing research careers [9]. Three to four months prior to the start of the program, we contact basic and clinical neuroscience research labs at McLean to ascertain who is interested and able to host an MMHRSP trainee for the 10-week program. MMHRSP mentors develop appropriate and well-crafted research projects for the trainees that are reviewed by the MMHRSP committee before the trainees begin, which ensures fit with the trainee’s interest and background, as well as feasibility within the program timeline. We emphasize that, ideally, the project will result in data that the trainee can present at SACNAS or ABRCMS and strongly encourage trainee co-authorship opportunities on any future manuscript(s). Once we have a critical mass of putative mentors and projects, trainees are matched to appropriate labs based on the interests listed on their applications. Throughout the 10-week program, the Program Committee meets several times with the mentors–both individually and as a group–to identify and address any questions or issues, and to generate suggestions for improvement if needed.

### Evaluation of the trainees’ experience

Prior to the creation of the Program, we contacted the institutional IRB to gain approval to collect and disseminate survey data. MMHRSP did not require IRB approval or oversight as defined by the regulations at 45 CFR 46.102 according to The Massachusetts General Brigham Institutional Review Board (IRB). It was designated a Quality Improvement/Program Evaluation status. We require that trainees and mentors complete surveys at the beginning and the end of the program to evaluate expectations (beginning) and experiences (end). Trainees also complete brief evaluations of all professional development programs. Using resources such as the Entering Research Curriculum [19], and a culturally responsive external evaluator, The Program Committee creates surveys to reflect and assess the goals of the program.

### Summary

**Fig. 3:**
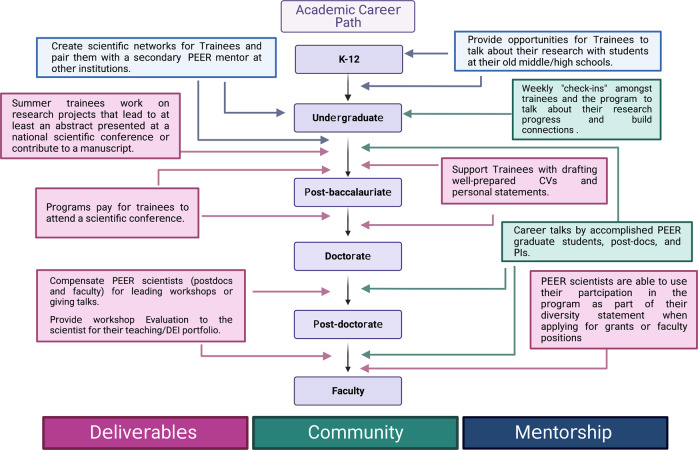
Creating an impactful summer research experience for the mental health careers of PEER individuals. Career path figure was influenced by Hinton AO, Jr, 2020 [1]. Figure was created with Biorender.

Summer research programs such as the MMHRSP provide a transformative opportunity for PEER trainees to experience hands-on research and build a strong foundation for a career in mental health research (Fig. 3). These opportunities provide students with scientific research experience, personal and professional guidance/mentorship, and fuel interest in mental health careers.

## References

[1] Medicine IO. In the Nation’s Compelling Interest: Ensuring Diversity in the Health-Care Workforce (2004). 2004, Washington DC: The National Academies Press. 25009857

[2] Jackson CS, Gracia JN (2014). Addressing health and health-care disparities: the role of a diverse workforce and the social determinants of health. Public Health Rep.

[3] Association, A.P. *Mental Health Disparities: Diverse Populations*. Available from: https://www.psychiatry.org/psychiatrists/cultural-competency/education/mental-health-facts.

[4] Programs, N.I.o.H.-D.i.E. *Underrepresented Racial and Ethnic Groups*. Available from: https://extramural-diversity.nih.gov/diversity-matters/underrepresented-groups.

[5] Chiao JY, Blizinsky KD (2013). Population disparities in mental health: insights from cultural neuroscience. Am J Public Health.

[6] Advisors M. Report of neuroscience departments & programs survey (Academic Year 2016–2017). 2017, Society for Neuroscience.

[7] Asai DJ (2020). Race Matters. Cell.

[8] Asai D. Excluded. J Microbiol Biol Educ. 2020;21. 10.1128/jmbe.v21i1.2071 PMC7148151 32313599

[9] Association, AP *CWS Data Tool: Demographics of the U.S. Psychology Workforce*. 2019; Available from: https://www.apa.org/workforce/data-tools/demographics.

[10] Wyse R, Hwang WT, Ahmed AA, Richards E, Deville C, Jr. Diversity by Race, Ethnicity, and sex within the US Psychiatry Physician Workforce. Acad Psychiatry. 2020;44:523–30. 10.1007/s40596-020-01276-z 32705570

[11] Hinton AO, Termini CM, Spencer EC, Rutaganira FUN, Chery D, Roby R (2020). Patching the Leaks: Revitalizing and Reimagining the STEM Pipeline. Cell.

[12] Pierszalowski S, Bouwma-Gearhart J, Marlow L (2021). A systematic review of barriers to accessing undergraduate research for STEM students: problematizing under-researched factors for students of color. Soc Sci.

[13] Quintana SM, Mahgoub L (2016). Ethnic and racial disparities in education: psychology’s role in understanding and reducing disparities. Theory Into Pract.

[14] Estrada M, Hernandez PR, Schultz PW. A Longitudinal Study of How Quality Mentorship and Research Experience Integrate Underrepresented Minorities into STEM Careers. CBE Life Sci Educ. 2018;17. 10.1187/cbe.17-04-0066 PMC6007776 29351912

[15] Hinton AO, Vue Z, Termini CM, Taylor BL, Shuler HD, McReynolds MR (2020). Mentoring minority trainees: Minorities in academia face specific challenges that mentors should address to instill confidence. EMBO Rep.

[16] in The Science of Effective Mentorship in STEMM, ML Dahlberg and A Byars-Winston, Editors. 2019: Washington (DC). 31958221

[17] Termini CM, Hinton AO, Jr., Garza-Lopez E, Koomoa DL, Davis JS, Martinez-Montemayor MM. Building Diverse Mentoring Networks that Transcend Boundaries in Cancer Research. Trends Cancer 2021;7:385–8. 10.1016/j.trecan.2021.01.001 PMC8062285 33563577

[18] Lessard L, Smith C, O’Connor S, Velasquez SE, Benson J, Garfield J, et al. Collaborative Assessment Of Collective Reach And Impact Among INBRE Supported Summer Undergraduate Research Programs Across The United States. J STEM Educ 2021;22:46–51. PMC8373203 34413711

[19] Gibbs KD, Jr., McGready J, Bennett JC, Griffin K. Biomedical Science Ph.D. Career Interest Patterns by Race/Ethnicity and Gender. PLoS One 2014;9:e114736. 10.1371/journal.pone.0114736 PMC4262437 25493425

[20] Gewin V (2020). The time tax put on scientists of colour. Nature.

[21] Branchaw JL, Butz AR, Smith AR. Entering Research - A Curriculum to Support Undergraduate and Graduate Research Trainees. Second ed. Navigating Research and Mentoring. 2020, New York: W.H. Freeman and Company.

